# Allometric scaling of the maximum metabolic rate of mammals: oxygen transport from the lungs to the heart is a limiting step

**DOI:** 10.1186/1742-4682-2-31

**Published:** 2005-08-11

**Authors:** Page R Painter

**Affiliations:** 1 Office of Environmental Health Hazard Assessment, California Environmental Protection Agency, P. O. Box 4010, Sacramento, California 95812, USA

## Abstract

**Background:**

The maximum metabolic rate (MMR) of mammals is approximately proportional to *M*^*0.9*^, where *M *is the mammal's body weight. Therefore, MMR increases with body weight faster than does the basal metabolic rate (BMR), which is approximately proportional to *M*^*0.7*^. MMR is strongly associated with the capacity of the cardiovascular system to deliver blood to capillaries in the systemic circulation, but properties of this vascular system have not produced an explanation for the scaling of MMR.

**Results:**

Here we focus on the pulmonary circulation where resistance to blood flow (impedance) places a limit on the rate that blood can be pumped through the lungs before pulmonary edema occurs. The maximum pressure gradient that does not produce edema determines the maximum rate that blood can flow through the pulmonary veins without compromising the diffusing capacity of oxygen. We show that modeling the pulmonary venous tree as a fractal-like vascular network leads to a scaling equation for maximum cardiac output that predicts MMR as a function of *M *as well as the conventional power function *aM*^*b *^does and that least-squares regression estimates of the equation's slope-determining parameter correspond closely to the value of the parameter calculated directly from Murray's law.

**Conclusion:**

The assumption that cardiac output at the MMR is limited by pulmonary capillary pressures that produce edema leads to a model that is in agreement with experimental measurements of MMR scaling, and the rate of blood flow in pulmonary veins may be rate-limiting for the pathway of oxygen.

## Introduction

The maximum metabolic rate (MMR) of mammals is measured as the rate of oxygen consumption during the maximum sustainable rate of exercise [1]. Unlike the basal metabolic rate (BMR), which consumes oxygen at rates far below the delivery capacity of the cardiovascular system [1,2], the MMR is largely determined by the maximal rate that the cardiovascular system can deliver oxygen to mitochondria in muscle tissue [1].

MMR has been measured in mammals ranging in size, *M*, from 0.007 kg (pygmy mice) to 575 kg (cattle). Regression of the logarithm of MMR (denoted *Q*) on the logarithm of *M *gives a maximum-likelihood estimate (MLE) of the exponent *b *in the allometric expression

*Q = aM*^*b *^    (1)

of 0.872 with a 95% confidence interval (CI) of 0.812–0.931 for MMR data from 32 mammalian species [1]. In contrast, regression analysis of BMR data from 619 mammalian species gives a MLE of the slope, 0.69, with 95% CI, 0.68–0.70 [3]

To explain the scaling of the metabolic rate in mammals, West *et al*. [4] and Bengtson and Eden [5] model the arterial network as a structure that starts with a single tube (aorta) that repeatedly branches into two (or more) smaller tubes. Branching continues until a tube (small arteriole) that supplies capillaries is reached. They assume that all paths from the heart to capillaries pass through *n *tubes and that the arterial network is a truncated self-similar fractal (*i.e*., a fractal-like network). The smallest vessels of the circulatory system have dimensions that vary little with body size, whereas the dimensions of the aorta and other great vessels are highly dependent on size. For convenience, we define level *1 *of the arterial tree (or venous tree) as the smallest arterioles (or venules). These have radius *r*_*1 *_and length *l*_*1*_. Each level *2 *vascular tube with radius *r*_*2 *_and length *l*_*2 *_is connected to *η*_*1 *_level *1 *structures. In general, each level *i+1 *tube of radius *r*_*i*+*1 *_and length *l*_*i*+*1 *_is connected to *η*_*i *_level *i *tubes. It follows from the assumption of a self-similar fractal that the branching ratio is a constant (denoted *η*) and that the ratio of tube lengths, *l*_*i*+*1*_/*l*_*i*_, is also a constant (denoted *L*) throughout the network.

The theory of West *et al*. minimizes the (pressure) × (volume) work of the heart that is required to pump one unit of blood against a difference in pressure equal to the pressure in the aorta minus the pressure in capillaries. This work per unit of blood flow is proportional to the impedance in the arterial network. Minimization of this energy cost for pulsatile flow in arteries is claimed to require area-preserving branching of the network (*i.e*., the ratio *r*_*i*+*1*_*/r*_*i*_, termed *R*, is equal to *η*^*1*/*2*^) and, as a consequence, to require that the density of capillaries in tissues is proportional to *M*^-*1*/*4 *^(assuming that the diameter of the aorta scales proportionally to *M*^*3*/*8 *^or that arterial blood volume scales proportionally to *M*). The theory's 3/4-power scaling prediction for metabolic rate follows from the assumption that metabolic rate is proportional to the total number of capillaries calculated as tissue capillary density multiplied by *M*, an assumption that is reasonable for MMR but not for BMR [1]. The theory of Bengtson and Eden assumes that energy dissipation per endothelial surface area is constant, leading to the conclusions that *R *is equal to *η*^*2*/*5 *^and that the total number of capillaries is proportional to *M*^*15*/*17 *^if the volume of blood in arteries scales proportionally to *M*. If it is assumed that the diameter of the aorta scales proportionally to *M*^*3*/*8*^, the number of capillaries is proportional to *M*^*15*/*16*^.

The scaling of the total number of capillaries in skeletal muscle, where over 90% of energy metabolism occurs during MMR exercise, is nearly identical to the scaling of MMR [1], and, as noted above, this scaling is not proportional to *M*^*3*/*4*^. The 95% CI for the scaling exponent for total capillary volume, 0.909 – 1.0559, contains 15/16 but not 3/4. Moreover, if either of these theories is adequate for predicting capillary density, it should correctly predict the scaling exponent for capillaries in the lung, which is 1.00 with 95% CI of 0.912 – 1.087 [6]. This CI contains 15/16 but not 3/4.

### A model for the maximum metabolic rate

While minimization of impedance does not by itself lead to a correct prediction of capillary density in muscle and lung tissue, it is clearly an important principle for design of mammalian vascular systems [7,8]. The potential importance of impedance is most apparent in the pulmonary venous circulation, where the entire output of the heart's right ventricle flows before blood enters the left atrium of the heart. The driving force for pulmonary venous return to the heart is the pressure at the venous end of pulmonary capillaries minus the diastolic pressure in the left atrium (denoted *P*_*LA*_).

The output of oxygen by the left ventricle of the heart into the aorta is equal to the input of oxygen from the lungs to the heart. This is equal to the cardiac blood output rate multiplied by the maximum amount of oxygen per ml of blood multiplied by the percent saturation of blood with oxygen. Pressure in the model is strictly increasing with flow. However, as pressure rises above oncotic pressure, interstitial edema increases and then more and more fluid accumulates within alveoli. Therefore, oxygen saturation is strictly decreasing as a consequence of the increasing barrier to oxygen diffusion from pulmonary air into capillaries. As a result, there is a blood flow rate, denoted *F*_*max*_, that produces the maximum uptake of oxygen in the lungs, which is also the maximum output of oxygen to the body. The pressure near the venous end of alveolar capillaries at *F*_*max *_is denoted Π_*max*_. Consequently, the pressure gradient that drives the return of blood in pulmonary capillaries back to the heart is

Δ*P*_*max *_= *F*_*max*_*I*_*p *_    (2)

where Δ*P*_*max *_= Π_*max *_- *P*_*LA *_and *I*_*p *_is the impedance of the pulmonary venous network. It is assumed that Π_*max *_is proportional to the oncotic pressure of blood, denoted Π_*o*_. The value of Π_*max *_is assumed to be approximately the same in mammals of different sizes because Π_*o *_appears to be nearly invariant in mammalian species, being approximately 20 mm Hg [9-11] and *P*_*LA *_is approximately 1 mm Hg. (All pressures in this article are measured relative to ambient pressure.) Therefore, the scaling of *F*_*max *_with body size depends largely on the scaling of *I*_*p*_.

The impedance of the pulmonary venous network is a consequence of its physical structure and the viscosity of blood (termed *ν*). The pulmonary arteries and veins form parallel fractal-like networks in each lung with arteries and veins of the same level having similar dimensions [12,13]. Small venules have dimensions that are body-size-invariant (*r*_*1 *_approximately 10^-5 ^m and *l*_*1 *_approximately 10^-4 ^m). These vascular tubes receive blood from the capillaries in pulmonary acini, the structures that comprise approximately 10,000 alveoli and that appear to be body-size-invariant in mammals [14].

The impedance of a fractal-like network is the sum of impedances contributed by each level of the network. We assume that the impedance *I*_*i *_due to level *i *is the value calculated from the Poiseuille theory for non-turbulent fluid flow, , where *N*_*i *_is the number of level *i *vessels [4]. Consequently, *I*_*i*+*1 *_is equal to . The observation that dimensions within acini are size-invariant leads to the conclusion that *η*, *R *and *L *are size-invariant in acini. We assume that these ratios remain constant throughout the network. Therefore, the factor *ηL/R*^*4 *^(denoted *α*) is assumed to be size-invariant, and the expression for *I*_*p *_is a geometric series (when *α *≠ 1) that simplifies to



Substitution of this formula into Equation (2) gives



The assumption that the acinus is a size-invariant structure implies that the number of level *1 *venules per acinus is independent of body size. Consequently, the total number of level *1 *venules, *N*_*1*_, is proportional to lung volume, which is proportional to body mass *M *[6]. The parameter *n *is the number of branchings from the pulmonary vein to level *1 *venules. Therefore *η*^*n *^= *N*_*1*_*∝ M*, which is written as *η*^*n *^= *M /M*_*1*_. The constant *M*_*1 *_is the mass of body tissue supplied with the oxygen in blood flowing through a single level *1 *venule. This is estimated to be approximately 10^-5 ^kg [15,16] leading to the equation *n = [log(M)-log(10*^*-5*^*)]/log(η)*. Substitution for *N*_*1 *_and *n *in Equation (4) gives *F*_*max *_= *KM/ [1-ζ*^*log(M)-log(0.00001)*^*]*, where *ζ *= *α*^*1/log(η) *^and *K *is the constant . The maximal rate oxygen uptake in the lungs, *Q*, is *U*_*o*_*F*_*max*_,, where *U*_*o *_is the oxygen uptake in the lungs per unit of blood. Therefore, when *α *≠ 1,

*Q = U*_*o*_*C M/ [1-ζ*^*log(M)-log(0.00001)*^*] *    (5)

where C is a constant. Note that *ζ *depends on the base used to define the logarithm. The base 10 is used in the following regression analysis. When *α *= 1, we have

*Q = U*_*o*_*C M/ [log(M)-log(0.00001)]/ log(η) *    (6)

Equation (5) is termed the general pulmonary venous flow capillary pressure model (PVFCP model), and Equation (6) is termed the constrained PVFCP model.

### Testing model predictions

**Figure 1:**
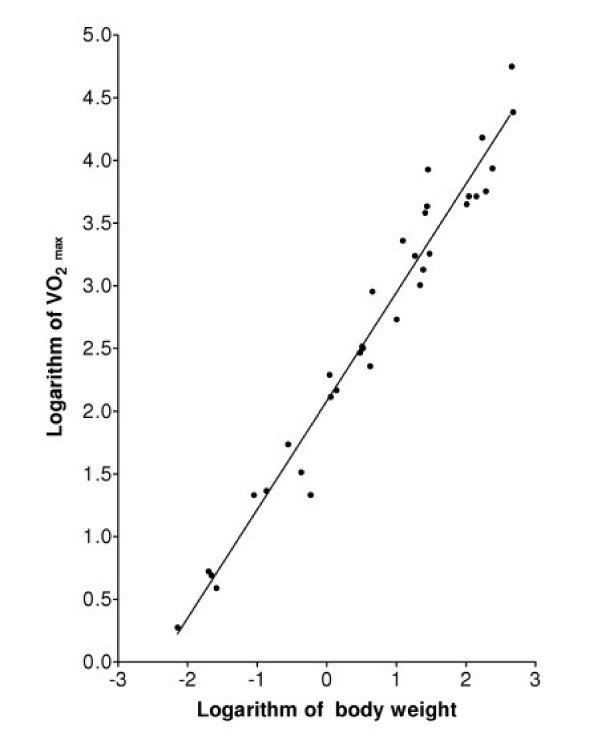
Regression analysis of MMR data in Table 1 (VO_2 _max in ml/min and body weight in kg) using the standard linear model, Equation (1). The minimum SSR is 1.6308.

**Figure 2:**
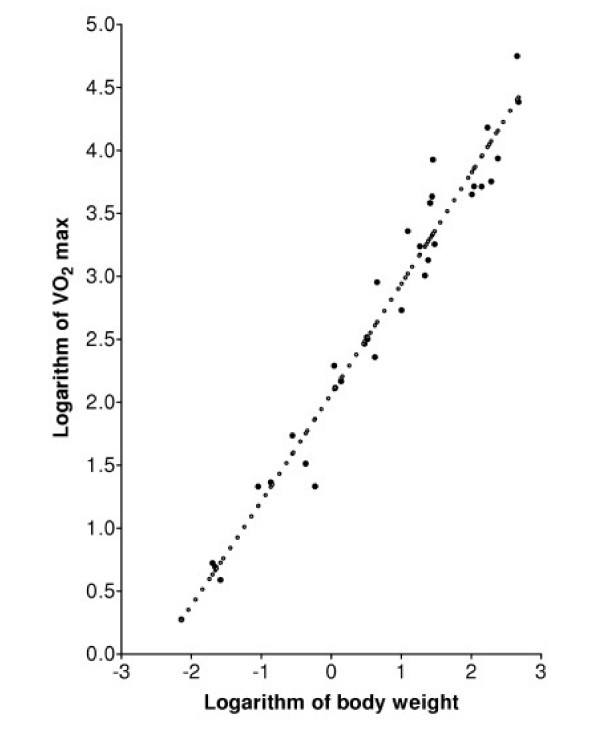
Regression analysis of MMR data in Table 1 (VO_2 _max in ml/min and body weight in kg) using the model of Equation (5). The closed circles are the data points from Table 1, and the open circles are the graph of the physiologically-based model, Equation (5), with parameters calculated from LSLR. The minimum SSR is 1.6263.

The conventional method for determining the best fit of Equation (5) or Equation (1) to oxygen uptake data is to find the values of the two parameters in the model that correspond to a minimum of the sum of squares of residuals (SSR), where a residual is defined as the logarithm of a measured value of the uptake rate minus the logarithm of the uptake rate predicted by the model for a mammal of the experimentally measured weight *M*. The technique is termed least squares logarithmic regression (LSLR). Figure 1 shows the best fit of the standard allometric model, Equation (1), to the data in Table 1. The minimal SSR occurs when *b *is 0.872 and the SSR is 1.6307. Figure 2 shows that the model of Equation (5), assuming that *U*_*o *_is constant, fits the data equally well: the minimal SSR occurs when the parameter *ζ*, which determines the slope of this scaling expression, is 1.193, and the SSR is 1.6269.

**Table 1:** Maximum metabolic rates (V_O2 _max) of mammals from Weibel *et al*.[1].

Mammal	M (kg)	V_O2 _max (ml/min)
Pygmy mouse	0.0072	1.884
Woodmouse	0.02	5.28
Deer mouse	0.022	4.928
Mouse	0.026	3.884
Chipmunk	0.09	21.485
Mole rat	0.136	14.58
Rat	0.278	23.13
Dwarf mongoose	0.43	54.44
Guinea pig	0.584	32.59
Rat kangaroo	1.1	194.7
Banded mongoose	1.14	130
Genet cat	1.38	146.6
Spring hare	3	291.6
Agouti	3.22	328.4
Suri	3.3	317.8
Dik-dik	4.2	228.1
Fox	4.51	897.5
Grant's gazelle	10.1	539.3
Coyote	12.4	2283.3
Pig	18.5	1731.6
African sheep	21.8	1013.7
Goat	24.3	1344.7
Dog	25.9	3825
Wolf	27.6	4310
Pronghorn	28.4	8435
Lion	30	1800
Wildebeest	102	4468
Waterbuck	110	5172
Calf	141	5161
Pony	171	15185
Zebu cattle	193	5660
Eland	240	8640
Horse	453	56005
Steer	475	24225

In the analysis of data in Table 1, it is assumed that maximum oxygen uptake is proportional to cardiac output (*i.e*. *U*_*o *_is constant). A more reasonable assumption is that oxygen uptake is proportional to cardiac output multiplied by the hemoglobin concentration of blood. The data in Table 2 include values of the hematocrit, which is nearly proportional to hemoglobin concentration. Therefore, the maximal rate of oxygen uptake multiplied by 0.42 and divided by the hematocrit (*i. e*., the oxygen uptake adjusted to a hematocrit of 0.42) is now assumed to be proportional to maximum cardiac output.

**Table 2:** Maximum metabolic rates of mammals adjusted to a standard hematocrit of 0.42 from Weibel *et al*.[1].

Mammal	Body mass (kg)	Hematocrit	V_O2 _max (ml/min)	
			Measured value	Adjusted value
Woodmouse	0.02	0.42	5.28	5.28
Mole rat	0.129	0.42	13.61	13.61
Rat	0.148	0.42	15.55	15.55
Guinea pig	0.595	0.5	33.2	27.888
Agouti	3.22	0.42	328.44	328.44
Fox	4.4	0.42	955.7	955.7
Goat	21	0.299	1386	1946.89
Dog	23.7	0.5	3455.5	2902.62
Pronghorn	28.4	0.456	8434.8	7768.895
Horse	446	0.55	60745.2	46387.24
Steer	475	0.4	24225	25436.25

LSLR using the data in Table 2 and the model of Equation (1) gives the value of 0.957 for *b *(R_c_^2 ^= .9697) and SSR = 0.5890) when the SSR is minimized. LSLR using Equation (5) finds that the SSR is minimized when *ζ *equals 0.801 (SSR = 0.5833). LSLR of predicted values of cardiac output from Equation (5) using values of *M *from Table 2 and the estimate for *ζ *of 0.801 gives *b *= 0.958 and R_c_^2 ^= 0.9991. Clearly the predictions from Equation (5) are again nearly indistinguishable from those of Equation (1), and Equation (5) fits these data as well as Equation (1) does.

**Figure 3:**
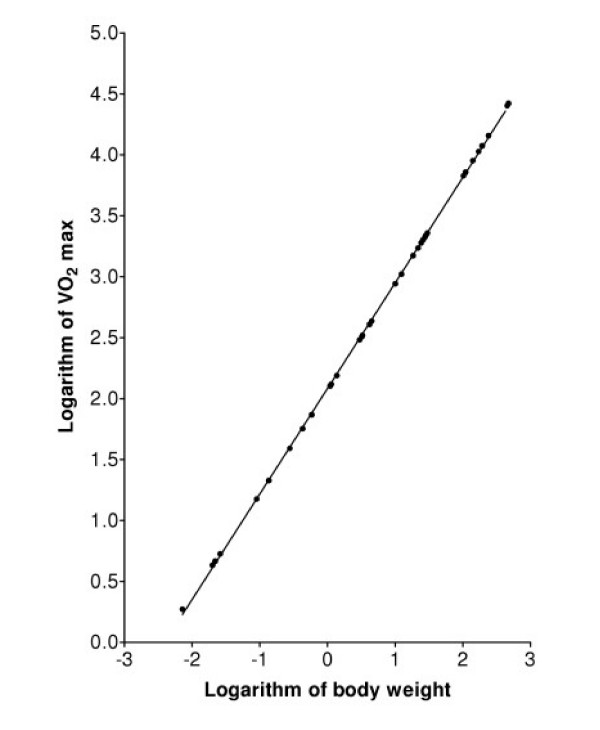
Predicted values of MMR from Equation (5) for mammals with the body weights in Table 1. The straight line is the best fit of the standard allometric model, Equation (1), to the predicted values.

While the logarithm of the function *Q *defined in Equation (5) is a nonlinear function of the logarithm of *M*, it is clear from Figure 2 that the logarithm of Q closely approximates a linear function of the logarithm of *M*. This observation is confirmed by substituting first-order approximations into Equations (5) and (6): The scaling of *Q *when *α *= 1 can be predicted directly from Equation (6). Multiplying and dividing by *log(M*_*1 *_*) *gives *Q *∝ *(M/log(M*_*1*_*))/(1 - log(M)/log(M*_*1*_*))*. Using logarithms to the base *e *and the first-order approximation *log*_*e*_*(1+x) = x *shows that log_e_(Q) is approximately equal to *log*_*e*_*(M) + log*_*e*_*(M)/log*_*e*_*(M*_*1*_*) *plus a constant *, i.e.*, *Q *is approximately proportional to *M*^*b *^where *b = 1 + 1/log*_*e*_*(M*_*1*_*)*. For *M*_*1 *_= 0.00001 *b *= 0.914, which is close to the value from LSLR of data simulated using Equation (6). A similar approximation analysis of Equation (5) shows that it too is approximately a power function when *α *is approximately equal to 1. Figure 3 shows that, with the parameters used in Figure 2, the logarithm of *Q *defined in Equation (5) is nearly identical to a linear function of the logarithm of *M*.

### Comparison with Murray's law

The estimate of *α = ηL/R*^*4 *^corresponding to *ζ *is *ζ*^*log(η)*^. For a branching ratio of 2 and *ζ *= 1.193, *α *is estimated to be 1.054. For a volume-filling fractal distribution network, it has been conjectured that [4]

*L = η*^*1/3*^,     (7)

and this equation for *L *leads to the formula

*R*^*3 *^= 1.04*η*.     (8)

Equation (8) is remarkably similar to Murray's law for the scaling of radii of arterial or venous networks, which states that flow rate is proportional to the third power of vessel radius [7]. For our network model, Murray's law implies *R*^*3 *^= *η*, and this equation together with the condition *L = η*^*1/3 *^implies *α *= 1. With this value of *α*, the slope of the logarithm of Equation (6) depends only on the estimate of *M*_*1*_. For *M*_*1 *_= 0.00001 kg, Equation (6) is nearly identical to a power function with *b *= 0.916. Therefore, Murray's law and the fractal length scaling relationship lead to the constrained PVFCP model and predict that the slope parameter of the scaling function is in the range of observed values.

## Discussion

The PVFCP model predicts that the logarithm of maximum oxygen uptake in mammals is approximately proportional to the logarithm of body mass. If the radii of veins in the pulmonary venous tree obey Murray's law, then the constant of proportionality is in the range of experimentally observed values for MMR. The PVFCP model, like other published explanations for MMR scaling, focuses on the supply of oxygen to the tissues. However, the PVFCP model differs from other explanations for MMR scaling because it focuses on pulmonary blood flow.

The PVFCP model and the model of Bengtson and Eden [5] use the same mathematical description of pressure-flow relationships in a vascular tree. While the model of Bengtson and Eden [5] is consistent with current data on MMR, the model's assumption of energy dissipation that is proportional to vascular surface area is questionable as a principle of mammalian design. For example, a hypothetical mammalian species that replaces the *R *= *η*^*2/5 *^requirement of their theory with the *R *= *η*^*1/3 *^relationship of Murray's law would reduce total energy dissipation in arteries. This replacement would also give a higher predicted capillary density and consequently a higher MMR.

It is instructive to compare the number of independent parameters and assumptions in the PVFCP model with the number of parameters and assumptions in the two fractal-like models of the arterial network that predict metabolic scaling [4,5]. All three models describe the vascular network as a self-similar fractal-like tubular structure with pressure gradients determined by Poiseuille's law. All assume that the size of terminal (smallest) network tubes is the same in mammals of different size and that blood viscosity does not vary with body size. All contain the branching ratio parameter *η *and the network length parameter *n*. In the PVFCP model, a relationship between *η*, *n *and body mass is derived from the assumption that the number of terminal segments is proportional to body mass, an assumption that is supported by observations. In the other two models, a relation between these parameters is derived from the assumption that arterial blood volume is proportional to body mass, an assumption without direct observational support. Network structure is related to metabolic rate in the PVFCP model by Equation (5), which specifies the maximum rate of blood flow that does not compromise pulmonary function. In the other models, such a relation is derived from the assumption that metabolic rate is proportional to the number of capillaries in the systemic circulation. In the PVFCP model, there is one more independent parameter, *α*, which is defined by fitting experimental data. The other models have two additional parameters, *L *and *R*. Both models specify *L *indirectly using the assumption of Equation (7). The parameter *R *is specified by an energy minimization principle in one model [4] and by an energy dissipation principle in the other [5]. While the number of parameters and assumptions in the PVFCP model is relatively large, it is less than the number in the fractal-like network models previously published. Another recent mathematical description of metabolic scaling, the "Allometric Cascade" model [2], is not discussed here because it is not a mechanistic model. Indeed, the two models appear compatible because the PVFCP model could be integrated into the "Allometric Cascade" model to provide a mechanism-based scaling term for the maximum rate of blood flow.

Weibel *et al*. [1] argue that it is the volume of mitochondria in muscle tissue and the blood supply in capillaries in muscle tissue that determine the scaling of MMR. This view is supported by their demonstration that MMR is remarkably correlated with and is proportional to mitochondrial volume (*b = 1.09, R*_*c*_^*2 *^= 0.9939) and to estimated capillary blood volume in muscle tissue (*b = 0.975, R*_*c*_^*2 *^= 0.9846). However, total mitochondrial volume and blood volume in muscle capillaries can be increased by exercise conditioning, and the correlation between capillary surface area and MMR or between mitochondrial volume and MMR may arise from such conditioning.

In the formulation of the PVFCP model, the role of gravity in facilitating or impeding the return of pulmonary blood to the heart has been ignored. Blood that is one inch higher than the left atrium has potential energy to facilitate its return to the heart that is approximately equivalent to a 2 mm Hg pressure gradient. For small mammals (*e.g*., mice), gravitational effects would be small compared with the approximately 20 mm Hg pressure gradient that we assume drives blood return during MMR exercise. However, for large mammals (e.g., elephants and whales), the effects of gravity will significantly increase blood return from regions of lung above the heart, but decrease blood return from regions below the heart. Therefore, Equation (5) may not adequately describe MMR blood flow in large mammals.

A second reason for doubting the validity of Equation (5) for large mammals is that intervals of the heart cycle increase with body size. The minimum length of the heart cycle (at maximum heart rate) is largely composed of the time required for the ventricles to fill plus the time required for the ventricles to eject blood into the pulmonary artery and aorta. At maximal heart rate, ventricular filling time is nearly equal to the PR interval, which is approximately proportional to the 1/4-power of body mass [17]. If the sum of the QRS interval and the ST segment, which is nearly equal to the time required to eject blood from the ventricles, has similar scaling, then the scaling exponent for maximum heart rate is less than the scaling exponent for the MMR divided by body mass, *i.e., *the specific maximum metabolic rate (SMMR). Thus, maximum heart rate, not the limitation posed by pulmonary venous impedance, may limit MMR for very large mammals.

The biological plausibility of the relation between MMR and *I*_*p *_proposed in the PVFCP model depends on whether pressures in lung capillaries approach the oncotic pressure of blood during periods of maximal exertion. In healthy humans at rest, the pressure difference between pulmonary capillaries and the left atrium ranges from approximately 5 to 11 mm Hg [18]. Assuming that the value of 5 mm Hg occurs when pulmonary veins are dilated, this pressure difference is predicted to increase by a factor of approximately 4 during heavy exercise in a trained athlete when cardiac output increases by a factor of 4 (assuming that the pulmonary veins are in a comparable state of dilation). This would require the capillary pressure to rise to approximately 21 mm Hg. It is noteworthy that signs of pressure stress are sometimes observed in pulmonary tissue from trained endurance athletes [19].

Studies of human patients with narrowing of the mitral valve, the valve between the left atrium and left ventricle, are consistent with the hypothesis that *I*_*p *_limits maximum metabolic rate. This condition, termed mitral stenosis, causes an increase in *P*_*LA*_. Patients with a *P*_*LA *_below 20 mm Hg usually do not have pulmonary edema at rest but may develop it with exercise. Furthermore, women with a *P*_*LA *_between 18 and 20 mm Hg are at risk for developing pulmonary edema during pregnancy where the cardiac output at rest increases on average by approximately 50% [20-22].

Additional support for the proposed role of pulmonary impedance in determining MMR comes from studies of horses, which have an MMR well above the value predicted by the allometric equation fitted to the data in Table 1[1]. Horses at rest have pulmonary capillary blood pressures that are above those in humans with mitral stenosis and pulmonary edema with exercise. Horses are apparently able to exercise without developing pulmonary edema because they are able to "concentrate" their blood during periods of exertion. The concentration of erythrocytes (measured as the hematocrit) is increased during exercise [23]. This requires a preferential loss of water that likely occurs in capillaries of the systemic circulation. As a result, the concentration of albumin in blood is increased and the oncotic pressure of blood is increased. This adaptation enables a horse at a gallop to tolerate pulmonary capillary pressures as high as 38 mm Hg [24].

Horses possess a second adaptation that allows them to increase their SMMR. Their ratio of lung volume to body mass is approximately 20% greater than the average value for mammals [6]. To pump blood through their large lungs at an unusually high rate per unit lung volume, horses possess a heart that is larger (as a fraction of body mass) than the average value for mammals [25]. This enables them to achieve a SMMR that is more than twice that of a cow of similar size. However, even with its remarkable adaptations, no horse can sustain the SMMR that pygmy mice and other small mammals can achieve [1].

## Competing interests

The author(s) declare that they have no competing interests.

## References

[B1] Weibel ER, Bacigalupe LD, Schmitt B, Hoppeler H (2004). Allometric scaling of maximal metabolic rate in mammals: muscle aerobic capacity as determinant factor. Respir Physiol Neurobiol.

[B2] Darveau C-A, Suarez RK, Andrews RD, Hochachka PW (2002). Allometric cascade as a unifying principle of body mass effects on metabolism. Nature.

[B3] White CR, Seymour RS (2003). Mammalian basal metabolic rate is proportional to body mass^2/3^. Proc Natl Acad Sci USA.

[B4] West GB, Brown JH, Enquist BJ (1997). A general model for the origin of allometric scaling laws in biology. Science.

[B5] Bengtson H-U, Eden P (2003). A simple model for the arterial system. J Theoret Biol.

[B6] Gehr P, Mwangi DK, Ammann A, Maloiy GM, Taylor CR, Weibel ER (1981). Design of the mammalian respiratoey system. V. Scaling morphometric pulmonary diffusing capacity to body mass: wild and domestic animals. Respir Physiol.

[B7] Murray CD (1926). The physiological principle of minimum work. I. The vascular system and the cost of blood volume. Proc Natl Acad Sci USA.

[B8] LaBarbera M (1990). Principles of design of fluid transport systems in zoology. Science.

[B9] Stohrer M, Boucher Y, Stangassinger M, Jain RK (2000). Oncotic pressure in solid tumors is elevated. Cancer Res.

[B10] Gabel JC, Scott RL, Adair TH, Drake RE, Traber DL (1980). Errors in calculated oncotic pressure of dog plasma. Am J Physiol.

[B11] Madigan JE, Rahal CJ (2003). Measurement of Plasma Colloid Osmotic Pressure in Normal Thoroughbred Neonatal Foals.

[B12] Agur AM, Ming JL, Grant JC (2004). Grant's Atlas of Anatomy.

[B13] Bergman RA, Afifi AK, Heidger PM (1996). Atlas of Microscopic Anatomy.

[B14] Weibel ER (1991). Fractal geometry: a design principle for living organisms. Am J Physiol.

[B15] Turcotte DL, Pelletier JD, Newman WI (1998). Networks with side branching in biology. J Theoret Biol.

[B16] Fung YC (1990). Biomechanics.

[B17] Noujaim SF, Lucca E, Munoz V, Persaud D, Berenfeld O, Meijler FL, Jalife J (2004). From mouse to whale: a universal scaling relation for the PR Interval of the electrocardiogram of mammals. Circulation.

[B18] Tabata T, Oki T, Fukuda N, Iuchi A, Manabe K, Kageji Y, Sasaki M, Yamada H, Ito S (1996). Influence of left atrial pressure on left atrial appendage flow velocity patterns in patients in sinus rhythm. J Am Soc Echocardiogr.

[B19] West JB (2004). Vulnerability of pulmonary capillaries during exercise. Exerc Sport Sci Rev.

[B20] Reis G, Motta MS, Barbosa MM, Esteves WA, Souza SF, Bocchi EA (2004). Dobutamine stress echocardiography for noninvasive assessment and risk stratification of patients with rheumatic mitral stenosis. J Am Col Cardiol.

[B21] Desai DK, Adanlawo M, Naidoo DP, Moodley J, Kleinschmidt I (2000). Mitral stenosis in pregnancy: a four-year experience at King Edward VIII Hospital, Durban, South Africa. BJOG.

[B22] van Oppen AC, Stigter RH, Bruinse HW (1996). Cardiac output in normal pregnancy: a critical review. Obstet Gynecol.

[B23] Weber J-M, Dobson GP, Parkhouse WS, Wheeldon D, Harman JC, Snow DH, Hochachka PW (1987). Cardiac output and oxygen consumption in exercising Thoroughbred horses. Am J Physiol.

[B24] Hackett RP, Ducharme NG, Gleed RD, Mitchell L, Soderholm LV, Erickson BK, Erb HN (2003). Do Thoroughbred and Standardbred horses have similar increases in pulmonary vascular pressures during exercise?. Can J Vet Res.

[B25] Hoppeler H, Lindstedt SL, Claassen H, Taylor CR, Mathieu O, Weibel ER (1984). Scaling mitochondrial volume in heart to body mass. Respir Physiol.

